# Without Data, There Is No Cure: Cancer Registries and Survival Intelligence as the Missing Link in WHO CureAll Implementation

**DOI:** 10.1590/0034-7167.20267903

**Published:** 2026-10-26

**Authors:** Luís Carlos Lopes-Júnior, Maurício Maza, Monnie Abraham, Regina Aparecida Garcia de Lima, María Liliana Vásquez Ponce

**Affiliations:** I Organização Pan-Americana da Saúde, Departamento de Doenças Não Transmissíveis e Saúde Mental. Washington, DC, United States of America; II St Jude Children’s Research Hospital, Departamento de Medicina Pediátrica Global. Memphis, Tennessee, United States of America; III Universidade de São Paulo, Escola de Enfermagem de Ribeirão Preto, Department of Maternal Infant Nursing and Public Health. Ribeirão Preto, São Paulo, Brazil

Childhood cancer remains one of the most inequitable conditions in global health^(1)^. While survival exceeds 80% in high-income countries, it is dramatically lower in lowand middle-income countries, where most children live and where health systems face persistent structural constraints. In response to these disparities, the World Health Organization, in collaboration with St. Jude Children’s Research Hospital and other global partners, launched the Global Initiative for Childhood Cancer (GICC) in 2018, with the ambitious goal of achieving at least 60% survival worldwide by 2030^(2)^.

The CureAll framework provides the operational architecture for this initiative, structured around four pillars-centers of excellence, universal health coverage, regimens for diagnosis and treatment, and evaluation and monitoring-supported by key enablers such as governance, financing, and advocacy^(2,3)^. While substantial progress has been made in expanding early diagnosis initiatives, strengthening clinical capacity, and improving access to essential medicines in the Americas, a critical dimension remains insufficiently addressed in both policy and practice: the systematic use of high-quality cancer data to guide decision-making and measure impact^(3)^.

Robust cancer registries-particularly population-based and hospital-based systems-are not merely technical tools; they are essential instruments of epidemiological surveillance and integral components of cancer care networks. As such, they must be fully embedded within the organization of health systems and care pathways, ensuring that data generation, monitoring, and evaluation align with service delivery and health system performance^(4)^. Without accurate and timely data on incidence, stage at diagnosis, treatment patterns, and survival, health systems operate in a context of partial visibility, limiting their ability to prioritize interventions, allocate resources efficiently, and evaluate outcomes^(3)^. In many countries in Latin America and the Caribbean, cancer registration systems remain fragmented, under-resourced, or incomplete, constraining the development of evidence-informed policies and masking the true magnitude of childhood cancer burden^(3,5)^. From this perspective, cancer registries should not be viewed as parallel systems, but as core elements of integrated care networks, supporting planning, coordination, and continuous improvement of services^(6)^.

The implications of these gaps are profound. Survival disparities across the region are not only a reflection of inequities in access to care but also of inequities in data systems^(1,3)^. Countries with stronger cancer registries are better positioned to identify delays in diagnosis, monitor treatment abandonment, evaluate quality of care, and implement targeted interventions^(4)^. Conversely, weak data infrastructures perpetuate invisibility, delaying action and reinforcing cycles of inequity^(4,5)^.

In this context, the concept of “survival intelligence” emerges as a strategic priority. Survival intelligence goes beyond the mere collection of data; it involves the integration, analysis, and real-time use of information to guide clinical practice, health system organization, and public policy. It encompasses the capacity to link datasets, generate survival estimates, identify disparities, and translate findings into actionable strategies. Within the CureAll framework, this aligns directly with the pillar of evaluation and monitoring, which explicitly calls for strengthening cancer registries and leveraging data systems to improve outcomes^(2)^.

Recent advances in the Americas demonstrate both the potential and the urgency of this approach. Regional initiatives coordinated by the Pan American Health Organization have expanded monitoring activities and promoted the development of country profiles and data systems aligned with CureAll priorities^(3,7)^. However, these efforts must be scaled and institutionalized to ensure sustainability and impact. Investments in cancer registries should be understood not as ancillary components but as core health system interventions, essential for achieving survival gains.

From a nursing and broader health workforce perspective, strengthening data systems also has critical implications^(8,9)^. Health professionals are central actors in data generation, documentation, and interpretation. Their engagement in data quality improvement, clinical audit processes, and implementation research is fundamental to building responsive and learning health systems. Moreover, integrating data literacy into professional training can empower frontline providers to use evidence in real time, improving patient navigation, adherence to treatment, and continuity of care^(10)^.

Importantly, advancing survival intelligence requires a shift in how success is measured. Beyond counting services delivered or professionals trained, health systems must prioritize outcome indicators-particularly survival and quality of life. This transition demands not only technical capacity but also political commitment to transparency, accountability, and continuous improvement.

Ultimately, achieving the CureAll target of 60% survival will depend not only on expanding access to care but also on the ability to measure, understand, and act upon outcomes. In the absence of reliable data, progress cannot be accurately assessed, and inequities remain hidden. Strengthening cancer registries and embedding survival intelligence within health systems is therefore not optional-it is indispensable.

As the Americas advance in GICC implementation, a clear message emerges: without data, there is no visibility; without visibility, there is no action; and without action, there is no cure. Investing in cancer registration and survival monitoring is, ultimately, an investment in equity, accountability, and the right of every child with cancer to survive and thrive.
