# Supplementary material for: Without Data, There Is No Cure: Cancer Registries and Survival Intelligence as the Missing Link in WHO CureAll Implementation
Source: Rev Bras Enferm. 2026 Oct 26;79:e7903. doi: 10.1590/0034-7167.20267903 (PMC13719131; doi:10.1590/0034-7167.20267903)
Supplement: Translation

# Translation: Sem Dados, Não Há Cura: Registros de Câncer e Inteligência em Sobrevida como o Elo Ausente na Implementação do *CureAll* da OMS

**DOI:** 10.1590/0034-7167.20267903pt

**Authors:** Luís Carlos Lopes-Júnior, Maurício Maza, Monnie Abraham, Regina Aparecida Garcia de Lima, María Liliana Vásquez Ponce

**Affiliations:** I Organização Pan-Americana da Saúde, Departamento de Doenças Não Transmissíveis e Saúde Mental. Washington, DC, Estados Unidos da América; II St Jude Children’s Research Hospital, Departamento de Medicina Pediátrica Global. Memphis, Tennessee, Estados Unidos da América; III Universidade de São Paulo, Escola de Enfermagem de Ribeirão Preto, Departamento de Enfermagem Materno-Infantil e Saúde Pública. Ribeirão Preto, São Paulo, Brasil

O câncer infantil permanece uma das condições mais desiguais na saúde global^(1)^. Enquanto a sobrevida ultrapassa 80% em países de alta renda, ela é significativamente menor em países de baixa e média renda, onde vive a maioria das crianças e onde os sistemas de saúde enfrentam restrições estruturais persistentes. Em resposta a essas disparidades, a Organização Mundial da Saúde, em colaboração com o *St. Jude Children’s Research Hospital* e outros parceiros globais, lançou a *Global Initiative for Childhood Cancer* (GICC) em 2018, com o ambicioso objetivo de alcançar pelo menos 60% de sobrevida em todo o mundo até 2030^(2)^.

O *framework CureAll* fornece a arquitetura operacional para essa iniciativa, estruturado em quatro pilares - centros de excelência, cobertura universal de saúde, regimes de diagnóstico e tratamento, e avaliação e monitoramento -, apoiados por facilitadores-chave como governança, financiamento e *advocacy* ^(2,3)^. Embora avanços substanciais tenham sido alcançados na expansão de iniciativas de diagnóstico precoce, no fortalecimento da capacidade clínica e na melhoria do acesso a medicamentos essenciais nas Américas, uma dimensão crítica permanece insuficientemente abordada tanto na política quanto na prática: o uso sistemático de dados de alta qualidade sobre câncer para orientar a tomada de decisão e medir impacto^(3)^.

Registros de câncer robustos, particularmente sistemas de base populacional e hospitalar, não são meramente ferramentas técnicas; são instrumentos essenciais de vigilância epidemiológica e componentes integrais das redes de atenção ao câncer. Como tal, devem estar plenamente incorporados à organização dos sistemas de saúde e aos fluxos assistenciais, garantindo que a geração, o monitoramento e a avaliação de dados estejam alinhados com a prestação de serviços e o desempenho do sistema de saúde^(4)^. Sem dados precisos e oportunos sobre incidência, estadiamento ao diagnóstico, padrões de tratamento e sobrevida, os sistemas de saúde operam com visibilidade parcial, limitando sua capacidade de priorizar intervenções, alocar recursos de forma eficiente e avaliar resultados^(3)^. Em muitos países da América Latina e do Caribe, os sistemas de registro de câncer permanecem fragmentados, subfinanciados ou incompletos, restringindo o desenvolvimento de políticas baseadas em evidências e obscurecendo a real magnitude da carga do câncer infantil^(3,5)^. Nessa perspectiva, os registros de câncer não devem ser vistos como sistemas paralelos, mas como elementos centrais de redes de atenção integradas, apoiando o planejamento, a coordenação e a melhoria contínua dos serviços^(6)^.

As implicações dessas lacunas são profundas. As desigualdades de sobrevida na região refletem não apenas inequidades no acesso ao cuidado, mas também desigualdades nos sistemas de informação^(1,3)^. Países com registros de câncer mais robustos estão mais bem posicionados para identificar atrasos no diagnóstico, monitorar abandono de tratamento, avaliar a qualidade do cuidado e implementar intervenções direcionadas^(4)^. Por outro lado, infraestruturas de dados frágeis perpetuam a invisibilidade, atrasando ações e reforçando ciclos de desigualdade^(4,5)^.

Nesse contexto, o conceito de “inteligência em sobrevida” emerge como uma prioridade estratégica. A inteligência em sobrevida vai além da simples coleta de dados; envolve a integração, análise e uso em tempo real das informações para orientar a prática clínica, a organização dos sistemas de saúde e as políticas públicas. Abrange a capacidade de integrar bases de dados, gerar estimativas de sobrevida, identificar desigualdades e traduzir achados em estratégias acionáveis. No *framework CureAll*, isso se alinha diretamente ao pilar de avaliação e monitoramento, que explicitamente prevê o fortalecimento dos registros de câncer e o uso de sistemas de dados para melhorar desfechos^(2)^.

Avanços recentes nas Américas demonstram tanto o potencial quanto a urgência dessa abordagem. Iniciativas regionais coordenadas pela Organização Pan-Americana da Saúde ampliaram as atividades de monitoramento e promoveram o desenvolvimento de perfis nacionais e sistemas de dados alinhados às prioridades do *CureAll* ^(3,7)^. No entanto, esses esforços precisam ser ampliados e institucionalizados para garantir sustentabilidade e impacto. Investimentos em registros de câncer devem ser compreendidos não como componentes acessórios, mas como intervenções centrais dos sistemas de saúde, essenciais para alcançar ganhos em sobrevida.

Sob a perspectiva da enfermagem e da força de trabalho em saúde de forma mais ampla, o fortalecimento dos sistemas de dados também possui implicações críticas^(8,9)^. Os profissionais de saúde são atores centrais na geração, documentação e interpretação dos dados. Seu engajamento na melhoria da qualidade dos dados, em processos de auditoria clínica e em pesquisas de implementação é fundamental para a construção de sistemas de saúde responsivos e baseados em aprendizagem. Além disso, a integração da literacia em dados na formação profissional pode capacitar os profissionais da linha de frente a utilizar evidências em tempo real, melhorando a navegação do paciente, a adesão ao tratamento e a continuidade do cuidado^(10)^.

É importante destacar que o avanço da inteligência em sobrevida exige uma mudança na forma como o sucesso é mensurado. Para além da contagem de serviços prestados ou profissionais capacitados, os sistemas de saúde devem priorizar indicadores de desfecho, especialmente sobrevida e qualidade de vida. Essa transição requer não apenas capacidade técnica, mas também compromisso político com transparência, responsabilização e melhoria contínua.

Em última análise, alcançar a meta do *CureAll* de 60% de sobrevida dependerá não apenas da ampliação do acesso ao cuidado, mas também da capacidade de medir, compreender e agir sobre os desfechos. Na ausência de dados confiáveis, o progresso não pode ser avaliado com precisão, e as desigualdades permanecem ocultas. Fortalecer os registros de câncer e incorporar a inteligência em sobrevida aos sistemas de saúde não é, portanto, opcional, mas indispensável.

À medida que as Américas avançam na implementação da GICC, uma mensagem clara emerge: sem dados, não há visibilidade; sem visibilidade, não há ação; e sem ação, não há cura. Investir em registro de câncer e monitoramento da sobrevida é, em última análise, investir em equidade, responsabilização e no direito de toda criança com câncer de sobreviver e prosperar.

# Translation: Sin Datos, No Hay Cura: Registros de Cáncer e Inteligencia de Supervivencia como el Eslabón Perdido en la Implementación de *CureAll* de la OMS

**DOI:** 10.1590/0034-7167.20267903es

**Authors:** Luís Carlos Lopes-Júnior, Maurício Maza, Monnie Abraham, Regina Aparecida Garcia de Lima, María Liliana Vásquez Ponce

**Affiliations:** I Organização Pan-Americana da Saúde, Departamento de Enfermedades No Transmisibles y Salud Mental. Washington, DC, Estados Unidos de América; II St Jude Children’s Research Hospital, Departamento de Medicina Pediátrica Global. Memphis, Tennessee, Estados Unidos de América; III Universidade de São Paulo, Escola de Enfermagem de Ribeirão Preto, Departamento de Enfermería Materno-Infantil y Salud Pública. Ribeirão Preto, São Paulo, Brasil

El cáncer infantil sigue siendo una de las condiciones más inequitativas en la salud global^(1)^. Mientras que la supervivencia supera el 80% en países de altos ingresos, es significativamente menor en países de ingresos bajos y medianos, donde vive la mayoría de los niños y donde los sistemas de salud enfrentan limitaciones estructurales persistentes. En respuesta a estas disparidades, la Organización Mundial de la Salud, en colaboración con *St. Jude Children’s Research Hospital* y otros socios globales, lanzó la *Global Initiative for Childhood Cancer* (GICC) en 2018, con el ambicioso objetivo de alcanzar al menos un 60% de supervivencia a nivel mundial para 2030^(2)^.

El *framework CureAll* proporciona la arquitectura operativa para esta iniciativa, estructurada en cuatro pilares - centros de excelencia, cobertura universal de salud, regímenes de diagnóstico y tratamiento, y evaluación y monitoreo -, respaldados por facilitadores clave como gobernanza, financiamiento y abogacía^(2,3)^. Si bien se han logrado avances sustanciales en la expansión de iniciativas de diagnóstico temprano, el fortalecimiento de la capacidad clínica y la mejora del acceso a medicamentos esenciales en las Américas, una dimensión crítica sigue insuficientemente abordada tanto en la política como en la práctica: el uso sistemático de datos de alta calidad sobre cáncer para orientar la toma de decisiones y medir el impacto^(3)^.

Los registros de cáncer robustos, particularmente los sistemas de base poblacional y hospitalaria, no son meramente herramientas técnicas; son instrumentos esenciales de vigilancia epidemiológica y componentes integrales de las redes de atención oncológica. Como tales, deben estar plenamente integrados en la organización de los sistemas de salud y en las rutas asistenciales, garantizando que la generación, el monitoreo y la evaluación de datos estén alineados con la prestación de servicios y el desempeño del sistema de salud^(4)^. Sin datos precisos y oportunos sobre incidencia, estadio al diagnóstico, patrones de tratamiento y supervivencia, los sistemas de salud operan con visibilidad parcial, lo que limita su capacidad para priorizar intervenciones, asignar recursos de manera eficiente y evaluar resultados^(3)^. En muchos países de América Latina y el Caribe, los sistemas de registro de cáncer siguen siendo fragmentados, subfinanciados o incompletos, lo que restringe el desarrollo de políticas basadas en evidencia y oculta la verdadera magnitud de la carga del cáncer infantil^(3,5)^. Desde esta perspectiva, los registros de cáncer no deben considerarse sistemas paralelos, sino elementos centrales de redes de atención integradas, que apoyan la planificación, la coordinación y la mejora continua de los servicios^(6)^.

Las implicaciones de estas brechas son profundas. Las disparidades en supervivencia en la región reflejan no solo inequidades en el acceso a la atención, sino también desigualdades en los sistemas de información^(1,3)^. Los países con registros de cáncer más sólidos están mejor posicionados para identificar retrasos en el diagnóstico, monitorear el abandono del tratamiento, evaluar la calidad de la atención e implementar intervenciones dirigidas^(4)^. Por el contrario, infraestructuras de datos débiles perpetúan la invisibilidad, retrasan la acción y refuerzan ciclos de inequidad^(4,5)^.

En este contexto, el concepto de “inteligencia de supervivencia” emerge como una prioridad estratégica. La inteligencia de supervivencia va más allá de la simple recopilación de datos; implica la integración, el análisis y el uso en tiempo real de la información para orientar la práctica clínica, la organización de los sistemas de salud y las políticas públicas. Incluye la capacidad de vincular bases de datos, generar estimaciones de supervivencia, identificar desigualdades y traducir los hallazgos en estrategias accionables. En el *framework CureAll*, esto se alinea directamente con el pilar de evaluación y monitoreo, que enfatiza el fortalecimiento de los registros de cáncer y el aprovechamiento de los sistemas de datos para mejorar los resultados^(2)^.

Los avances recientes en las Américas demuestran tanto el potencial como la urgencia de este enfoque. Iniciativas regionales coordinadas por la Organización Panamericana de la Salud han ampliado las actividades de monitoreo y promovido el desarrollo de perfiles de país y sistemas de datos alineados con las prioridades de *CureAll* ^(3,7)^. Sin embargo, estos esfuerzos deben ampliarse e institucionalizarse para garantizar su sostenibilidad e impacto. Las inversiones en registros de cáncer deben entenderse no como componentes secundarios, sino como intervenciones centrales de los sistemas de salud, esenciales para lograr mejoras en la supervivencia.

Desde la perspectiva de la enfermería y de la fuerza laboral en salud en general, el fortalecimiento de los sistemas de datos también tiene implicaciones críticas^(8,9)^. Los profesionales de la salud son actores clave en la generación, documentación e interpretación de datos. Su participación en la mejora de la calidad de los datos, en auditorías clínicas y en investigaciones de implementación es fundamental para construir sistemas de salud resilientes y orientados al aprendizaje. Además, integrar la alfabetización en datos en la formación profesional puede empoderar a los profesionales de primera línea para utilizar evidencia en tiempo real, mejorando la navegación del paciente, la adherencia al tratamiento y la continuidad del cuidado^(10)^.

Es importante destacar que avanzar en la inteligencia de supervivencia requiere un cambio en la forma de medir el éxito. Más allá de contar servicios prestados o profesionales capacitados, los sistemas de salud deben priorizar indicadores de resultados, especialmente la supervivencia y la calidad de vida. Esta transición exige no solo capacidad técnica, sino también compromiso político con la transparencia, la rendición de cuentas y la mejora continua.

En última instancia, alcanzar el objetivo de *CureAll* de un 60% de supervivencia dependerá no solo de ampliar el acceso a la atención, sino también de la capacidad de medir, comprender y actuar sobre los resultados. En ausencia de datos confiables, el progreso no puede evaluarse con precisión y las inequidades permanecen ocultas. Fortalecer los registros de cáncer e integrar la inteligencia de supervivencia en los sistemas de salud no es opcional: es indispensable.

A medida que las Américas avanzan en la implementación de la GICC, emerge un mensaje claro: sin datos, no hay visibilidad; sin visibilidad, no hay acción; y sin acción, no hay cura. Invertir en el registro de cáncer y en el monitoreo de la supervivencia es, en última instancia, invertir en equidad, rendición de cuentas y en el derecho de cada niño con cáncer a sobrevivir y prosperar.
